# Faking Orgasm: Relationship to Orgasmic Problems and Relationship Type in Heterosexual Women

**DOI:** 10.1016/j.esxm.2021.100419

**Published:** 2021-08-20

**Authors:** Krisztina Hevesi, Zsolt Horvath, Dorottya Sal, Eszter Miklos, David L. Rowland

**Affiliations:** 1 Institute of Psychology, ELTE Eötvös Loránd University, Budapest, Hungary; 2 Doctoral School of Psychology, ELTE Eötvös Loránd University, Budapest, Hungary; 3 Department of Psychology, Valparaiso University, Valparaiso IN USA

**Keywords:** Faking Orgasm, Pretending Orgasm, Women, Orgasmic Difficulty, Relationship Types, One-Night Stands

## Abstract

**Introduction:**

Although faking orgasm among women appears quite common, the roles of orgasmic problems and relationship type in partnered sex and masturbation have not been fully explored.

**Aim:**

We assessed the relationship between orgasmic problems (separately in partnered sex and masturbation) and faking orgasm across various types of relationships while also considering the role of demographic and sexual function related covariates.

**Methods:**

In this study of 1,168 Hungarian women, we assessed orgasmic problems, sexual functioning, and the prevalence and frequency of faking orgasm in 3 relationship types: romantic relationships, one-night stands, and continuing sexual relationships of more than one night.

**Main Outcome Measure:**

Differences in faking orgasm across 3 types of relationships; the association between faking orgasm in 1 type of relationship with faking orgasm in other types of relationships; and the relationship of “orgasmic problems” in partnered sex and masturbation to the presence and frequency of faking orgasm in various relationship types.

**Results:**

A subset of women who faked orgasm in 1 relationship type were more likely to fake orgasm in other relationship types. Orgasmic problems predicted the occurrence and frequency of faking orgasm in all 3 relationship types, though to different degrees. Other factors, including orgasmic difficulty during masturbation, the frequencies of masturbation and partnered sex, and the self-rated importance of sex were also related to the frequency of faking orgasm.

**Conclusion:**

Our findings suggest that faking orgasm has both dispositional and situational elements. “Orgasmic problems” in partnered sex was a consistent and robust predictor of the occurrence and frequency of faking orgasm in different relationship types.

**Hevesi K, Horvath Z, Sal D, et al. Faking Orgasm: Relationship to Orgasmic Problems and Relationship Type in Heterosexual Women. Sex Med 2021;9:100419.**

## INTRODUCTION

The prevalence of faking orgasm (or “feigning” or “pretending” orgasm) among women appears to be quite common, with early research indicating that about 55% of women had faked orgasm at least once in their lifetime.^1^^,^^2^ This general pattern has since been confirmed in more recent studies, some reporting rates as high as 67–74% while others suggesting more moderate rates around 30–40%.^3^, ^4^, ^5^, ^6^, ^7^, ^8^, ^9^, ^10^

Explanations for faking orgasm have been related to many factors, including dispositional (internal), situational (external), and contextual (eg, relationship types and issues).^1^^,^^4^^,^^11^^,^^12^ Among the more common *internal* motivations for faking orgasm among women are: avoiding negative feelings related to not reaching orgasm, realizing orgasm is unlikely, and attempting to heighten their sexual arousal.^3^^,^^13^, ^14^, ^15^, ^16^ Beliefs about gender and sexuality may also play a role in motivating women to fake orgasm: those who endorse anti-feminist values and hostile sexism are more likely to fake orgasm than others.^17^ Similarly, so are women who believe that female orgasm is necessary for men to fulfill their own gratification and pleasure needs.

Other explanations for faking orgasm have focused on *situational* factors such as fatigue and exhaustion, boredom with the sexual activity, wanting to end intercourse, and intoxication.^4^^,^^16^^,^^18^ The *context* of the sexual intercourse and the type of the relationship also appear to influence women's perceived need to fake orgasm. Men in committed relationships attend more to pleasing their partners, whereas both men and women in hook ups (ie, relationships defined as primarily or exclusively sexual in nature) are more likely to question women's, though not men's, right to pleasure.^19^ This gender disparity in behaviors and assumptions across relationship types may result in women more likely faking orgasm in a hookup than, say, in a long-term relationship. Furthermore, in longer term relationships, consideration of the partner typically assumes greater importance, with such factors as protecting the partner's self-esteem, avoiding partner disappointment, living up to expectations, maintaining partner interest and arousal, retaining the partner and avoiding infidelity, and avoiding conflict and unpleasant conversations playing a greater role.^7^, ^8^, ^9^^,^^11^^,^^13^, ^14^, ^15^^,^^20^^,^^21^ As specific examples, 1 study reported that 78% of respondents faked orgasm to avoid conflict or to spare their partner's feelings, and 47% to please their partner.^18^ Another study has shown that faking orgasm is closely tied to the woman's assessment of the importance of her orgasm to her (male) partner as well as her worries about the effects of her lack of orgasm on him.^22^^,^^23^ Furthermore, relationships characterized by mutual care and commitment typically involve a greater variety of sexual activities, thus increasing the woman's likelihood of orgasm^19^ and lessening the need to fake it.

## ORGASMIC DIFFICULTY AS A MOTIVATOR FOR FAKING ORGASM

One of the more intuitive factors thought to be related to faking orgasm is having difficulty reaching orgasm during partnered sex (orgasmic difficulty: OD). The prevalence of women struggling to reach orgasm during partnered sex somewhat parallels that of faking orgasm, with estimates ranging between 35% and 60%.^24^, ^25^, ^26^, ^27^ Consistent with this idea, women who have difficulty reaching orgasm are more likely to have faked orgasm over the course of their lifetime.^17^

Thus, faking orgasm may be 1 way for women to conceal their OD and avoid shame, embarrassment, or feelings of failure during partnered sex.^3^^,^^9^^,^^12^^,^^14^^,^^21^^,^^24^^,^^28^^,^^29^ Yet, while several studies have supported a positive relationship between OD and faking orgasm,^3^^,^^9^^,^^17^^,^^28^, ^29^, ^30^ broad consensus surrounding its role as a primary reason is lacking. For example, 1 study has demonstrated that although faking orgasm and orgasmic disorders correlate positively, the percentage of explained variance is typically low, suggesting the relevance of other variables.^5^ Another has reported that sexual dysfunction tended to play a secondary role in faking orgasm, with some women who faked orgasm not necessarily lacking orgasmic capacity during partnered sex.^13^ Furthermore, relationship type is to some extent associated with relationship/sexual satisfaction,^31^ which has shown a reciprocal relationship with OD: Reaching orgasm not only affects the evaluation of the relationship,^32^ but poor relationship satisfaction likely increases arousal difficulty and OD.^15^^,^^33^, ^34^, ^35^ Thus, OD, relationship type, and faking orgasm may all be interrelated.

## RATIONALE AND AIMS

Although OD is 1 variable correlated with faking orgasm, other factors are important to consider. As just noted, the propensity toward faking orgasm may vary across situations, including the specific type of relationship, where the woman's investment in, or her assessment of potential for, a continuing relationship may vary (eg, one-night hook-up vs romantic relationship). In addition, the role of other sexual response parameters, including the importance of sex to the woman, her orgasmic capacity in general as assessed during masturbation, and her overall frequency of sexual activity have not been evaluated in conjunction with the putative relationship between OD and faking orgasm. Such variables are relevant because they broaden the understanding of other contextual factors that might be related to faking orgasm.

Given the above, this exploratory analysis considered 3 questions: (1) Do the presence and rate of faking orgasm differ across various relationship types (eg, higher in one-night stands or continuing sexual relationships than romantic relationships) (Aim 1)? (2) To what extent is faking orgasm in 1 type of relationship (presence and/or frequency) related to faking orgasm in other types of relationships (that is, are these related or independent phenomena) (Aim 2)? (3) Does having OD during partnered sex and/or masturbation increase the likelihood of faking orgasm and, furthermore, do known covariates of OD (ie, importance of sex, frequency of partnered sex and masturbation) and/or key demographic variables increase that likelihood (Aim 3)?

## MATERIAL AND METHODS

### Participants and Procedure

Participants from Hungary were recruited for an online cross-sectional study using 3 strategies: (1) invitations to local university students as 1 way to earn extra credit in their courses; (2) postings on Facebook (social media site); and (3) invitations via articles on sexual psychological themes in online Hungarian magazines. Before accessing the questionnaire, participants gave informed consent, declared being at least 18 years old, and acknowledged the voluntary and anonymous nature of the study as well as the option to terminate participation at any time without consequence. Ethical approval for the study was obtained from the Institutional Review Boards of the authors’ institutions.

Overall, 2220 individuals responded to the survey. Analysis was limited to self-verified cisgender, “primarily/exclusively” heterosexual women having a sexual partner and having ever masturbated in their lifetime, resulting in a final sample of 1168 women. Specifically, separate questions were used to determine participants’ sex and gender (ie, responses of “female” were required on both items for inclusion in the analyses), and respondents who indicated bisexual, primarily homosexual, or asexual orientations were excluded. The requirement of “having ever masturbated” was included for 2 reasons: (1) orgasmic problems during partnered sex do not provide an overall estimate of orgasmic capacity, which can be better assessed through masturbation,^36^, ^37^, ^38^ and (2) the role of this variable in faking orgasm in partnered sex has not previously been investigated. Sample characteristics are provided in Table 1.

**Table 1:** Sample characteristics and prevalence of faking orgasm

Recruitment source N (%)	
Via university courses	230 (19.7%)
Via Facebook, or friends	444 (38.0%)
Via online magazines	491 (42.1%)
Other source	2 (0.2%)
Age	
M (SD)	32.84 (10.76)
Range (Minimum – Maximum)	50 (18–68)
Level of education N (%)	
Less than high-school graduation	10 (0.9%)
High-school graduation	446 (38.2%)
Vocational education (after high-school graduation)	192 (16.4%)
University or college degree	496 (42.5%)
Postgraduate degree	23 (2.0%)
Faked orgasm ever in a romantic relationship N (%)	
Have not ever had a romantic relationship	18 (1.5%)
Have had a romantic relationship but not ever faked orgasm	438 (37.5%)
Have ever faked orgasm in a romantic relationship	711
% of total sample	60.9%
% of subset ever in a relationship [95% CI]	61.9% [59.1–64.6%]
Faked orgasm ever in a one-night sexual relationship N (%)	
Have not ever had a one-night relationship	459 (39.4%)
Have had a one-night relationship but not ever faked orgasm	438 (37.6%)
Have ever faked orgasm in a one-night relationship	269
% of total sample	23.1%
% of subset in a one-night relationship [95% CI]	38.0% [34.5–41.6%]
Faked orgasm ever in a sexual relationship continuing > one night N (%)	
Have not ever had a sexual relationship lasting more than one night	476 (40.9%)
Have had a continuing sexual relationship but not ever faked orgasm	408 (35.0%)
Have ever faked orgasm in a continuing sexual relationship	281
% of total sample	24.1%
% of subset in a continuing sexual relationship (>1 night) [95% CI]	40.8% [37.1–44.5%]

### Measures

#### Presence and Frequency of Faking Orgasm in Different Relationship Types

Faking orgasm was assessed within the context of 3 sexual relationship types: (1) in romantic relationships, defined as any relationship characterized by more than just sexual interactions between partners, that is, including other relationship dimensions such as romantic feelings towards the partner; (2) in one-night stands, defined as a primarily or exclusively sexual relationship that occurred only in a single night; and (3) in continuing sexual relationships lasting more than 1 night, defined as a primarily or exclusively sexual relationship which continued beyond a single night.

For each of these 3 relationship types, 2 questions assessed faking orgasm. The first assessed the presence/absence of faking orgasm in the specific relationship type (henceforth referred to as “ever” faking orgasm in that relationship type). Then, for those who indicated that “yes,” they had faked an orgasm in that relationship type, the frequency of faking orgasm was assessed on a 10-point scale (1 = almost never to 10 = Always).

#### Orgasmic and Sexual Functioning

Multiple items measured orgasmic and sexual functioning during partnered sex and masturbation. Items were selected from a larger 42-item questionnaire measuring dimensions of sexual, orgasmic, and relationship functioning.^34^^,^^36^^,^^37^^,^^39^ 4 items measured orgasmic response separately during either partnered sex (based on their current or most recent relationship) or masturbation (during the past 9–12 months). Specifically, women estimated (1) the percent of time they reached orgasm relative to their overall sexual episodes (1 = Never, 10 = Always), (2) difficulty reaching orgasm (0 = Always reaching orgasm, 5 = Nearly always [having difficulty reaching orgasm]), (3) orgasmic latency (1 = 1–5 minutes [to reach orgasm], 7 = I do not reach orgasm), and (4) orgasmic pleasure (1 = Very satisfying, 6 = Do not reach orgasm). Because these variables showed moderate to high correlations within each type of sexual activity (|r| = 0.58–0.68 for partnered sex and |r| = 0.47–0.58 for masturbation), 2 composite latent variables were created to represent *orgasmic problems* during each type of sexual activity, partnered sex and masturbation. The 4 orgasmic indicators used to generate these composite latent variables were treated as ordered categorical variables. Previous research has used this same approach to combine sets of related variables into single composite measures of overall/general level of orgasmic problems during partnered sex and (separately) masturbation to achieve a more precise measurement (eg, less bias from measurement error; the composite measure is not dependent on the response scale of the orgasmic variables).^38^ Both composite variables presented satisfactory internal consistency (orgasmic problems in partnered sex: α = 0.84; orgasmic problems in masturbation: α = 0.79).

Single-item questions also assessed the importance of sex (1 = Not important at all, 5 = Very important), and the frequency of partnered sex and, separately, masturbation (1 = almost never, 8 = one or more times daily) over the past 9–12 months.

Reliability analysis for a number of survey items assessing sexual response, including ones used in this analysis, had been carried out on a subset of 424 participants, with test-retest correlations ranging from 0.64 to 0.85 (median value, 0.71), thus showing an overall moderate level of reliability.^34^^,^^37^^,^^39^

### Data Analysis

Data analysis involved a 2-step process. First, we assessed bivariate relationships of “ever” faking orgasm or the frequency of faking orgasms across the various types of relationships. Then, in the second set of regression analyses, we predicted “ever” faking orgasm and the frequency of faking orgasm for each relationship type (romantic, one-night, and continuing sexual), using orgasmic problems, related sexuality parameters (described above), and relevant demographic variables as predictor covariates. Each of these steps is described in greater detail in the following paragraphs.

#### Bivariate Associations

In the first analytical step, bivariate associations between measures of faking orgasm across different types of relationships were calculated. Specifically, associations between *ever* faking orgasm across different relationship types (0 = absence, 1 = presence of faking orgasm) were estimated for the subset of women reporting each of the relationship types. Chi-square (χ^2^) statistics were calculated as tests of hypothesis and phi correlations (φ) were calculated to assess effect sizes. In order to investigate these relationships further, we also generated concordance rates (ie, the proportion of those women who reported ever faking orgasm in both relationship types) and odds ratios with 95% confidence intervals (ie, whether faking orgasm in either type of relationship is associated with higher odds of also faking orgasm in the other type of relationship). Then, Spearman correlations between the *frequency of faking orgasm* across different relationship types were estimated, again using only the subset of women who reported having both relationship types *and* faking orgasm in those relationship types.

#### Regression Analyses

In the second analytical step, multiple regression was used to identify predictors of faking orgasm in the different types of relationships, first for *ever* faking orgasm by using binary probit regression, then for the *frequency* of faking orgasm by using ordered categorical probit regression within the specific relationship type. Specifically, the first outcome variable—*ever* faking orgasm—was represented by a dichotomous, categorical variable (0 = absence, 1 = presence of faking orgasm: Models 1a–3a); and the second outcome variable—*frequency* of faking orgasm—was represented by an ordered categorical outcome variable (1 = almost never to 10 = always) and included only those women who reported ever faking orgasm in that relationship type (Models 1b–3b). For all models, predictor variables included: recruitment source, age, level of education, importance of sex, frequency of partnered sex, frequency of masturbation, and the 2 composite latent variables related to orgasmic problems for (1) partnered sex and (2) masturbation.

All models were estimated using the Weighted Least Squares Mean and Variance method with the probit link function. Similar to binary and multinomial logistic regression models, the probit link function can be used in regression models having dichotomous (ie, presence vs absence of faking orgasm) or ordered categorical (ie, frequency of faking orgasm) outcome variables. However, for regression models that use the probit link function to estimate the parameters, the relationships between the predictor and outcome variables are usually represented by standardized Beta (β) regression coefficients (having values between ±1.00, with interpretation similar to correlation estimates) rather than by odds ratios. As the regression models contained latent predictor variables which were defined by ordered categorical variables (ie, *orgasmic problems* during partnered sex and masturbation), the use of the Weighted Least Squares Mean and Variance estimation method was preferred over maximum likelihood estimation (ie, logistic regression models). Analyses used SPSS (IBM Corp. Released 2019. IBM SPSS Statistics for Windows, Version 26.0 Armonk, NY) and Mplus 8.0 statistical software.^40^ Data supporting the included analyses are available from the corresponding author upon reasonable request.

## RESULTS

### Aims 1 &2: Presence/Absence and Frequency of Faking Orgasm Across Relationship Types

Rates (and confidence intervals [CI]) of *ever* faking orgasm in different relationship types are presented in Table 1. For the subsets of women reporting each specific relationship type, *ever* faking orgasm was 61.9% in romantic relationships (*P* < .05 with the other 2 types); 40.8% in continuing relationships; and 38% in one-night stands.

Bivariate associations between *ever* faking orgasm across different types of relationships are shown in Table 2*.* Specifically, *ever* faking orgasm in a romantic relationship showed significant, moderate, and positive associations with *ever* faking orgasm in continuing sexual relationships and *ever* faking orgasm in one-night stands. In addition, a significant, strong and positive relationship occurred between *ever* faking orgasm in continuing sexual relationships and *ever* faking orgasm in one-night stands. In line with these, in each of the bivariate associations, *ever* faking orgasm in either type of relationship was associated with higher odds of also *ever* faking orgasm in the other types of relationships, represented by significant ORs ranging from 5.33 (between *ever* faking orgasm in romantic relationships and one-night stands) to 9.22 (between *ever* faking orgasm in one-night stands and continuing sexual relationships). Concordance rates ranged from 29.4% (between *ever* in continuing sexual and one-night relationships) to 36.6% (between *ever* in romantic and continuing relationships).

**Table 2:** Bivariate associations between *ever* faking orgasm across different types of relationships

	Ever faked orgasm in romantic relationships	Ever faked orgasm in one-night stands	Ever faked orgasm in continuing sexual relationships
Ever faked orgasm in romantic relationship	XXX	CR = 32.90% (N = 230) OR [95% CI] = 5.33 [3.58–7.95]	CR = 36.62% (N = 249) OR [95% CI] = 8.76 [5.66–13.56]
Ever faked orgasm in one-night stand	χ^2^(1) = 75.75 (*P* < .001) φ = 0.33	XXX	CR = 29.41% (N = 150) OR [95% CI] = 9.22 [6.13–13.85]
Ever faked orgasm in continuing sexual relationship	χ^2^(1) = 115.04 (*P* < .001) φ = 0.41	χ^2^(1) = 127.29 (*P* < .001) φ = 0.50	XXX

This table shows associations between ever faking orgasm (0 = absence, 1 = presence) for pairs of relationship types. Below the diagonal are Chi-square statistics (χ^2^) (for test of hypothesis) and phi correlations (φ) (as effect-size measure). Above the diagonal are concordance rates (CR) and odds ratios (OR) with 95% confidence intervals [95% CI], representing the level of co-occurrence of faking orgasm across pairs of relationship types. CR represent the proportion of those women who reported ever faking orgasm in both types of relationships. OR represent the odds for ever faking orgasm in both relationship types.

Bivariate associations between the *frequency* of faking orgasm across different relationship types are shown in Table 3. The frequency of faking orgasm in continuing sexual relationships showed significant, strong, and positive correlations with frequencies of faking orgasm in one-night stands and in romantic relationships. The frequency of faking orgasm in one-night stands was significantly, positively, but only weakly associated with frequency of faking orgasm in a romantic relationship.

**Table 3:** Bivariate associations (Spearman r_s_) between frequency of faking orgasm (1 = almost never to 10 =always) across different types of relationships

	Frequency of faking orgasm in romantic relationships	Frequency of faking orgasm in one-night stands	Frequency of faking orgasm in continuing sexual relationships
Frequency of faking orgasm in romantic relationships	XXX	r_s_ = 0.20 (*P* = .003)	r_s_ = 0.51 (*P* < .001)
Frequency of faking orgasm in one-night stands		XXX	r_s_ = 0.58 (*P* < .001)
Frequency of faking orgasm in continuing sexual relationships			XXX

### Aim 3: Predictors of Faking Orgasm in Each Type of Relationship

#### Predictors of Faking Orgasm in Romantic Relationships (Models 1a and 1b)

Table 4 identifies significant predictors of *ever* faking orgasm and the *frequency* of faking orgasm in romantic relationships. *Ever* faking orgasm in romantic relationships was predicted by higher orgasmic problems during partnered sex, higher frequency of masturbation, lower importance of sex, and being recruited via Facebook or online magazines. Higher *frequency* of faking orgasm for the subset of women who ever faked orgasm in a romantic relationship was predicted by higher orgasmic problems in partnered sex and lower orgasmic problems in masturbation, and also not having a university/college degree. The strong relationship between orgasmic problems during partnered sex and frequency of faking orgasm was responsible for the higher level of explained variance in Model 1b compared with Model 1a.

**Table 4:** Predictors of *ever* faking orgasm and (separately) frequency of faking orgasm in romantic relationships (Models 1a & 1b), in one-night stands (Models 2a & 2b), and in continuing sexual relationships (Models 3a & 3b)

	Model 1a (N = 1114): Ever faked orgasm in romantic relationships		Model 1b (N = 691): Frequency of faking orgasm in romantic relationships		Model 2a (N = 684): Ever faked orgasm in one-night relationships		Model 2b (N = 256): Frequency of faking orgasm in one-night relationships		Model 3a (N = 665): Ever faked orgasm in continuing sexual relationships		Model 3b (N = 272): Frequency of faking orgasm in continuing sexual relationships	
Outcome variables	β (SE)	p	β (SE)	P	β (SE)	p	β (SE)	P	β (SE)	p	β (SE)	p
Recruitment source*: Facebook	**0.19 (0.06)**	**0.002**	0.04 (0.06)	0.496	**0.19 (0.08)**	**0.023**	0.10 (0.12)	0.429	0.15 (0.09)	0.097	0.14 (0.10)	0.139
Recruitment source*: Online magazines	**0.18 (0.06)**	**0.005**	0.03 (0.06)	0.646	0.12 (0.08)	0.164	0.13 (0.12)	0.293	0.08 (0.09)	0.363	0.13 (0.09)	0.153
Age	0.08 (0.05)	0.090	0.04 (0.04)	0.397	0.04 (0.06)	0.480	-0.08 (0.07)	0.253	0.03 (0.05)	0.531	-0.11 (0.07)	0.094
Level of education†	-0.05 (0.04)	0.185	**-0.09 (0.04)**	**0.024**	**-0.11 (0.05)**	**0.034**	0.08 (0.07)	0.214	-0.09 (0.05)	0.072	0.04 (0.06)	0.453
Importance of sex	**-0.09 (0.04)**	**0.032**	0.04 (0.04)	0.265	-0.07 (0.05)	0.198	**-0.15 (0.06)**	**0.012**	0.00 (0.05)	0.951	0.07 (0.05)	0.215
Frequency of partnered sex	0.07 (0.04)	0.115	-0.01 (0.04)	0.875	-0.03 (0.05)	0.516	**0.17 (0.06)**	**0.007**	-0.04 (0.05)	0.432	**0.21 (0.06)**	**0.001**
Frequency of masturbation	**0.09 (0.04)**	**0.036**	-0.05 (0.04)	0.213	0.04 (0.05)	0.422	-0.04 (0.07)	0.597	0.04 (0.05)	0.462	**-0.18 (0.06)**	**0.003**
Orgasmic problems in partnered sex	**0.10 (0.05)**	**0.033**	**0.50 (0.04)**	**<0.001**	0.04 (0.07)	0.531	**0.24 (0.09)**	**0.006**	**0.20 (0.07)**	**0.002**	**0.57 (0.08)**	**<0.001**
Orgasmic problems in masturbation	0.09 (0.06)	0.114	**-0.11 (0.04)**	**0.009**	-0.08 (0.07)	0.230	0.00 (0.09)	0.994	-0.08 (0.07)	0.233	-0.13 (0.07)	0.051
Explained variance (R^2^)	6.3%		23.9%		4.5%		9.4%		6.8%		26.9%	

⁎ Reference category: recruitment via university courses.

† Coded as 0 = Graduated at high-school, vocational education or absence of high-school graduation, 1 = Graduated at university or college. Standardized regression coefficients (β) and related standard error (SE) and *P* values in bold indicate *P* < .05. Predictors of ever faking orgasm (Models 1a–3a:) were determined using binary probit regression, while the predictors of the frequency of faking orgasm (Models 1b–3b) were determined using ordered categorical probit regression models.

#### Predictors of Faking Orgasm in One-Night Relationships (Models 2a and 2b)

Table 4 identifies predictors of *ever* faking orgasm and the *frequency* of faking orgasm in one-night stands. 2 predictors were significant for *ever* faking orgasm in such relationships: those participants recruited via Facebook (compared to women from university courses) and the absence of university or college degree were more likely to fake orgasm. Higher *frequency* of faking orgasm for the subset of women who ever faked orgasm in one-night stands was associated with lower importance of sex, as well as higher frequency of sex and higher orgasmic problems during partnered sex.

#### Predictors of Faking Orgasm in Continuing Sexual Relationships (Models 3a and 3b)

Table 4 identifies predictors of *ever* faking orgasm and the *frequency* of faking orgasm in continuing sexual relationships. *Ever* faking orgasm was predicted only by higher orgasmic problems during partnered sex. However, *higher frequency* of faking orgasm was predicted by higher frequency of sex, higher orgasmic problems during partnered sex, and lower frequency of masturbation. The relationship of orgasmic problems during partnered sex to frequency of faking orgasm was strong, which contributed to higher explained variance in Model 3b compared to Model 3a.

## DISCUSSION

This analysis enabled a unique perspective on orgasm faking by women. It allowed comparisons across types of relationships; identified predictors of faking orgasm, including orgasmic problems during partnered sex; and specified several covariates relevant to the relationship between orgasmic problems and faking orgasm.

### Implications of Faking Orgasm across Relationship Types

The prevalence of *ever* faking orgasm in a romantic relationship was 62%, a rate in the upper midrange of the prevalence continuum reported by various studies.^3^, ^4^, ^5^, ^6^, ^7^, ^8^, ^9^, ^10^ The prevalences for one-night stands and sexual relationships continuing beyond one-night were both around 40%, not only demonstrating the relatively high rate of faking orgasm across a variety of relationship types, but also shedding possible light on the range of prevalences reported previously across various studies.

Correlations and concordance rates suggest that some women may be more prone to faking orgasm, no matter the type of relationship. Specifically, concordance rates for *ever* faking orgasm across relationship types hovered around 30–35%, and moderately strong bivariate associations occurred for both *ever* and the *frequency* of faking orgasm across various relationship types. Thus, it appears that a subset of women who fake orgasm in 1 type of relationship were more likely to do so in other types of relationships—for these women, the tendency to fake orgasm seems more dispositional than situational/contextual, consistent with the idea that “personality type” may play a role in faking orgasm.^5^ Such dispositional factors may include any number of internally-derived characteristics, including, for example, women's attachment style,^41^ avoiding negative feelings about not reaching orgasm, self-focused reasons related to enhancing arousal,^42^ and various beliefs and attitudes.^3^^,^^13^, ^14^, ^15^, ^16^, ^17^

### Predictors of Faking Orgasm

#### Role of Orgasmic Problems

Orgasmic problems during partnered sex significantly predicted *ever* faking orgasm in continuing sexual relationships and in romantic relationships, but not in one-night stands. As noted previously,^19^ a woman's right to pleasure may not be assumed by either the man or woman in one-night hook ups, and therefore the motivation for women to fake orgasm may be diminished in such situations. Indeed, among college women, the motivations for non-committal sex are as tied to relationship seeking and social enhancement as to pleasure seeking^43^^,^^44^ whereas in continuing sexual or romantic relationships, women's motivations to fake orgasm may be linked to communication difficulties stemming from embarrassment about the topic or not wanting to hurt their partner's feelings.^45^ In this respect, contextual factors such as relationship type appear to play an important role in understanding whether women faked orgasms, and furthermore, in whether OD was a prominent factor related to its frequency. Interestingly, once a woman has faked orgasm in any specific relationship type, OD becomes the strongest and most consistent predictor of the *frequency* of faking orgasm, lending considerable support to the purported relationship between these 2 factors.^5^^,^^17^

### Other Sexual Factors Predicting the Occurrence and Frequency of Faking Orgasm

Although having orgasmic problems was by far the strongest and most consistent predictor of the frequency of faking orgasm in all relationship types, other predictors emerged as well. For example, the frequency of both partnered sex and masturbation were also relevant. The more partnered sex the woman reported, the greater her frequency of faking orgasm in one-night stands and ongoing sexual relationships—not surprising as greater frequency of sex suggests greater reason/opportunity for faking orgasm. The role of masturbation, however, was more complex: having orgasmic problems during masturbation—indicating a problem with overall orgasmic *capacity* rather than just diminished ability to reach orgasm during partnered sex—was related to lower frequency of faking orgasm in romantic relationships, suggesting that women who struggled to reach orgasm in masturbation simply realized that orgasm was likely out of reach for them during partnered sex (eg, ^9^^,^^12^^,^^21^^,^^24^^,^^29^). On the other hand, women who masturbated more frequently showed a lower frequency of faking orgasm in continuing sexual relationships but were more likely to ever do so in romantic relationships. That is, women's propensity for masturbating was related to their propensity for faking orgasm in different types of relationships. Although no simple explanation is apparent for this differential effect of masturbation on romantic vs continuing sexual relationships, it may be related to women's greater motivation to align masturbation stimulation with partnered sex activities in ongoing relationships focused primarily on sexual interaction and pleasure.^37^^,^^39^ Whatever the case, such results attest to the value of assessing women's total sexual activities in order to better understand variance in orgasm faking across different relationship types. Finally, the higher the woman's self-rated importance of sex, the less frequently she faked orgasm in romantic and one-night relationships. Though not a strong predictor overall, some women (eg, those in both types of relationships) may use their *lack* of reaching orgasm as a way of communicating to their partner that they have *not* been sexually satisfied by the encounter; alternatively, some women may rate sex as important (eg, in a romantic relationship) for reasons other than sexual pleasure, for example, for the feelings of intimacy and shared physicality that it affords.^46^^,^^34^^,^^37^

#### Role of Demographic Covariates

While education level and recruitment source were occasionally linked to the occurrence and frequency of faking orgasm, effect sizes were generally small. One interesting pattern was that women who were more educated were less likely to fake orgasm in romantic relationships or in one-night stands, a finding that contrasts with other research suggesting the inverse relationship.^6^ In addition, women who were recruited via Facebook or online magazines (vs students attending university) were more likely to ever have faked orgasm in a one-night stand or romantic relationship. Age and education differences might have accounted for this disparity, as well as differences in motivations for participation: college students were offered an incentive for participation whereas others—lacking such incentives—may have been motivated to volunteer because they viewed the topic as one of interest and/or relevance to their own situations. Nevertheless, significant findings related to demographic variables such as education should be interpreted cautiously due not only to their relatively weak effect size in this analysis but also to the use of convenience sampling which may over- or under-represent specific demographic subgroups of respondents (eg, those having greater access to social/public media). Representative sampling is needed to explore possible mechanisms underlying significant demographic covariates on faking orgasm, for example, examining the possible mediator role of psychosocial characteristics such as beliefs about gender and sex.^17^

### Implications and Conclusions

This study indicates a robust and consistent association between orgasmic problems and faking orgasm in women, both its occurrence and frequency. In addition, results suggest that both dispositional and contextual factors are relevant to understanding when and how frequently women fake orgasm. Specifically, substantial concordance occurred in faking orgasm across different relationship types, suggesting a dispositional influence; yet the prevalence of faking orgasm, as well as its predictors, differed to some extent across relationship types, suggesting a contextual influence. Other factors, including frequency of masturbation, general orgasmic capacity (as assessed by orgasmic capacity during masturbation), and importance of sex were also relevant to understanding faking orgasm, reiterating the complex and multivariate nature of this phenomenon. Understanding the above patterns not only provides insight into situations that encourage specific sexual scripts during sexual encounters, but also suggests the importance of communication, reciprocal caring and pleasure, and feelings of self-efficacy in longer term dyadic relationships.

## STRENGTHS AND LIMITATIONS

Our study included the benefits common to many online/non-online surveys:^47^ a sizable sample drawn from Hungary and anonymity afforded through an Internet approach which reduces social desirability and improves openness in responding.^48^^,^^49^ At the same time, our conclusions were limited by several factors. First, a potential for systematic bias within the sample stemmed from the social media recruitment strategy for some participants. Second, although we asked participants to respond to 3 distinct relationship types, overlap across types was possible, for example, a continuing sexual relationship might have morphed into a romantic relationship—thus relationship categories were not always mutually exclusive. Third, the cross-sectional nature of the study prevented conclusions regarding a causal effect of OD on faking orgasm—it may be that those women who habitually fake orgasm deprive themselves of an opportunity to learn the skill set (eg, both in terms of partner communication and stimulation adjustment) important for increasing orgasmic ability during partnered sex.^39^ Fourth, we lacked specific information regarding the duration of the relationships, as such information would have enabled inclusion of such covariates in the analysis. Fifth, the assessment instrument for faking orgasm was not standardized. The use of validated psychometric tests related to faking orgasm, including ones designed to specifically assess orgasmic parameters (eg,^50^), might strengthen the validity of response items in the future.

As our analysis was limited to cisgender heterosexual women and occurred within a highly specific Western context using a WEIRD (white, educated, industrialized, rich, democratic) sample,^51^ future studies investigating the role of cultural (non-Western), educational, and sexual (gender and orientation) diversity in faking orgasm are needed. Novel directions might also consider examining variables related specifically to the characteristics and descriptions of women's orgasms, for example, whether specific typologies of orgasm (clitoral, vaginal, ejaculatory, etc.), as well as women's beliefs about types of orgasms, might be related to women's likelihood and/or frequency of faking orgasm.^52^

## Informed Consent

Informed consent was obtained from all individual participants included in the study.

## Institutional Review Board Statement

The study was conducted according to the guidelines of the Declaration of Helsinki, and approved by the Ethics Committees (IRB) of Valparaiso University, USA, and Eötvös Loránd University, Hungary.

## Data Availability Statement

Data can be made available upon reasonable request.

## STATEMENT OF AUTHORSHIP

Krisztina Hevesi: Conceptualization, Methodology, Data Collection and Curation, Formal Analysis, Writing—Review and Editing; Zsolt Horvath: Methodology, Data Collection and Curation, Formal Analysis, Writing—Original Draft Preparation, Writing—Review And Editing; Dorottya Sal: Data Collection and Curation, Investigation and Literature Review, Writing—Original Draft Preparation, Writing—Review and Editing; Eszter Miklos: Methodology, Data Collection and Curation, Investigation and Literature Review, Writing—Original Draft Preparation, Writing—Review and Editing; David L. Rowland: Conceptualization, Methodology, Data Collection and Curation, Formal Analysis, Investigation and Literature Review, Writing—Original Draft Preparation, Writing—Review and Editing.

All authors have read and agreed to the published version of the manuscript.
